# Impaired spatial memory and enhanced long-term potentiation in mice with forebrain-specific ablation of the *Stim* genes

**DOI:** 10.3389/fnbeh.2015.00180

**Published:** 2015-07-14

**Authors:** Gisela Garcia-Alvarez, Mahesh S. Shetty, Bo Lu, Kenrick An Fu Yap, Masatsugu Oh-Hora, Sreedharan Sajikumar, Zoë Bichler, Marc Fivaz

**Affiliations:** ^1^Program in Neuroscience and Behavioral Disorders, Duke-NUS Graduate Medical SchoolSingapore, Singapore; ^2^Department of Physiology, Yong Loo Lin School of Medicine, National University of SingaporeSingapore, Singapore; ^3^Division of Molecular Immunology, Medical Institute of Bioregulation, Kyushu UniversityHigashi-ku, Japan; ^4^Behavioral Neuroscience Laboratory, National Neuroscience InstituteSingapore, Singapore

**Keywords:** excitatory synapse, AMPA receptor, endoplasmic reticulum, PKA, spatial memory, long-term potentiation, STIM1 and STIM2

## Abstract

Recent findings point to a central role of the endoplasmic reticulum-resident STIM (Stromal Interaction Molecule) proteins in shaping the structure and function of excitatory synapses in the mammalian brain. The impact of the *Stim* genes on cognitive functions remains, however, poorly understood. To explore the function of the *Stim* genes in learning and memory, we generated three mouse strains with conditional deletion (cKO) of *Stim1* and/or *Stim2* in the forebrain. *Stim1*, *Stim2*, and double *Stim1/Stim2* cKO mice show no obvious brain structural defects or locomotor impairment. Analysis of spatial reference memory in the Morris water maze revealed a mild learning delay in *Stim1* cKO mice, while learning and memory in *Stim2* cKO mice was indistinguishable from their control littermates. Deletion of both *Stim* genes in the forebrain resulted, however, in a pronounced impairment in spatial learning and memory reflecting a synergistic effect of the *Stim* genes on the underlying neural circuits. Notably, long-term potentiation (LTP) at CA3-CA1 hippocampal synapses was markedly enhanced in *Stim1/Stim2* cKO mice and was associated with increased phosphorylation of the AMPA receptor subunit GluA1, the transcriptional regulator CREB and the L-type Voltage-dependent Ca^2+^ channel Cav1.2 on protein kinase A (PKA) sites. We conclude that STIM1 and STIM2 are key regulators of PKA signaling and synaptic plasticity in neural circuits encoding spatial memory. Our findings also reveal an inverse correlation between LTP and spatial learning/memory and suggest that abnormal enhancement of cAMP/PKA signaling and synaptic efficacy disrupts the formation of new memories.

## Introduction

STIM1 and STIM2 are endoplasmic reticulum (ER)-resident Ca^2+^ sensors that regulate store-operated Ca^2+^ entry (SOCE), the major Ca^2+^ influx pathway in non-excitable cells (Liou et al., 2005; Roos et al., 2005). A recent body of evidence indicates that STIMs also function in the brain and shape synaptic transmission and architecture (Gruszczynska-Biegala et al., 2011; Hartmann et al., 2014; Sun et al., 2014; Garcia-Alvarez et al., 2015). Although both STIM isoforms are expressed throughout the nervous system, STIM1 is more abundant in the cerebellum (Skibinska-Kijek et al., 2009; Hartmann et al., 2014), while STIM2 is the predominant isoform in the hippocampus (Berna-Erro et al., 2009; Skibinska-Kijek et al., 2009; Sun et al., 2014; Garcia-Alvarez et al., 2015), an expression pattern which is consistent with the reported functions of these isoforms in the brain. STIM1 controls mGluR1-dependent synaptic transmission in Purkinje neurons and cerebellar motor behavior (Hartmann et al., 2014), whereas STIM2 influences dendritic spine morphogenesis (Sun et al., 2014; Garcia-Alvarez et al., 2015) and regulates AMPA receptor (AMPAR) phosphorylation and trafficking in the hippocampus (Garcia-Alvarez et al., 2015). Notably, STIM2 has also been associated with brain pathologies. Systemic ablation of the *Stim2* gene protects against hypoxic neuronal cell death (Berna-Erro et al., 2009) and reduced levels of STIM2 in a presenilin-1 mouse model of Alzheimer's disease accounts for a decrease in the fraction of mature (mushroom) dendritic spines in hippocampal neurons (Sun et al., 2014).

Most studies on STIM function in the brain report an effect of these genes on neuronal Ca^2+^ homeostasis (Hartmann et al., 2014; Sun et al., 2014), Ca^2+^ influx (through SOCE) (Berna-Erro et al., 2009; Sun et al., 2014) or Ca^2+^ mobilization from intracellular stores (Hartmann et al., 2014), suggesting that the STIM proteins regulate synaptic function primarily through their ability to shape synaptic Ca^2+^ concentration. Results from our laboratory recently highlighted a novel, SOCE-independent function of STIM2 in excitatory neurons in coupling the AMPAR subunit GluA1 to PKA (Garcia-Alvarez et al., 2015), indicating that STIMs may impact synaptic transmission through multiple signaling pathways. An interesting feature of STIMs is their redistribution to contact sites between the ER and the plasma membrane (PM) in response to lowering of ER Ca^2+^ (Liou et al., 2005; Roos et al., 2005; Wu et al., 2006) or cAMP elevation in the cytoplasm (Tian et al., 2012; Garcia-Alvarez et al., 2015). Dynamic localization to ER-PM contact sites allow STIMs to interact with and modify the properties of PM channels and receptors both in excitable and non-excitable cells (Park et al., 2009, 2010; Wang et al., 2010; Garcia-Alvarez et al., 2015). Together, these findings highlight an unprecedented level of crosstalk between the ER and the synapse and suggest that ER-to-synapse signaling sculpts functional neural circuits in the brain.

The impact of the *Stim* genes on behavior remains, however, largely unexplored. Systemic deletion of *Stim1* or *Stim2* is lethal within days or weeks after birth (Oh-Hora et al., 2008; Berna-Erro et al., 2009), precluding behavioral phenotyping of adult mice. Here, we report the behavioral analysis of *Stim1*, *Stim2*, and double *Stim1/Stim2* cKO mice, in which the *Stim* genes are selectively excised from the forebrain after birth. While single gene deletion has no or little effect on cognitive functions, ablation of both *Stim* genes leads to severe deficits in spatial learning and memory. Surprisingly, double *Stim1/Stim2* cKO mice exhibit enhanced LTP and elevated PKA signaling in the hippocampus, suggesting that uncontrolled PKA activity and synaptic strength deleteriously affects the acquisition of spatial memories.

## Materials and methods

### Mouse lines

*Stim1^fl/fl^*, *Stim2^fl/fl^*, and double *Stim1^fl/fl^/Stim2^fl/fl^* mice, first described in Oh-Hora et al. (2008), were crossed to B6.Cg-Tg(*CaMKIIα-Cre*)T29-1Stl/J (Tsien et al., 1996a) provided by the Jackson Laboratory (JAX, stock # 005359). The tdTomato Cre reporter line Ai9 (Madisen et al., 2010) was obtained from JAX (stock # 007909). The *Stim^fl/fl^*, *CaMKIIα-Cre* and Ai9 lines are all derived from the C57BL/6J background. All animal procedures were approved by the Institutional Animal Care and Use Committee (IACUC) of Singapore.

### Immunohistochemistry and confocal microscopy

Four-month-old mice were anesthetized with pentobarbital and transcardially perfused with phosphate-buffered saline (PBS), followed by 4% paraformaldehyde (PFA) in PBS, pH 7.4. Brains were dissected out, post-fixed in 4% PFA at 4°C overnight and cryoprotected for the next 2 days in 15 and 30% sucrose at 4°C. 40 μm coronal sections were cut on a freezing microtome and stored in cryoprotectant (30% ethylene glycol, 30% glycerol in PBS) at −20°C. For immunostaining, free floating sections were permeabilized with 0.25% Triton X-100 in PBS for 30 min and blocked with 2% BSA, 10% horse serum and 0.25% Triton X-100 in PBS for 1 h at room temperature. Sections were incubated with chicken anti-MAP2 antibody (1:1500, AbCam ab5392) at 4°C overnight, followed by incubation with goat anti-chicken 488 secondary antibody (Alexa Fluor, Life Technologies) for 1 h at room temperature and 5 μM Hoechst 33342 for 15 min. Sections were then mounted on glass slides with 0.2% gelatine in 50 mM Tris-HCl (pH 7.5), and covered with FluorSave (CalBiochem) using coverslips. Slides were imaged on a Zeiss LSM 710 inverted laser scanning confocal microscope using a 10X air objective. The Hoechst and MAP2 channels were excited with 405 and 488 nm solid-state lasers respectively. The full view of coronal sections was acquired using the Tile Scan function of the Zen 2010 B software.

### Behavioral analysis

All behavioral tests were conducted on 3–3.5 month old males. Experiments were performed blind to genotype.

#### Open field test (OFT)

Mice were placed in the middle of a closed arena (45 × 45 cm) and their activity was monitored for 15 min using the ANY-maze™ videotracking system (Stoelting, USA). The following parameters were scored: distance traveled, time in the center, rearing, grooming, and immobility, as previously described in Branchi et al. (2004).

#### Elevated plus maze (EPM)

The gray colored metal alloy maze consists of a 60 cm elevated plus-shaped apparatus with four arms (35 cm length × 5 cm width), two of them surrounded with walls of 15 cm height (Ugo Basile, Italy). Mice were placed on the central platform facing a closed arm and were allowed to freely explore the maze for 5 min. Behavior was automatically analyzed with the ANY-maze™ videotracking system, as described in Bichler et al. (2013).

#### Morris water maze (MWM)

The MWM paradigm was adapted from Manns et al. (2010). Each mouse was placed in a 120 cm diameter gray circular pool filled with 40 cm deep opaque water and containing a fixed hidden (submerged) platform. The animals were tested four times a day for five consecutive days, with an inter-trial of about 20 min. For each trial, mice were released facing the tank wall from one randomly selected starting point (North, South, East, or West), and were allowed to swim until they reach the platform (latency) or for a maximum of 60 s. On the 6th day, a 60 s probe trial was conducted without platform and the time spent in each quadrant was analyzed. Reversal learning was initiated 1 h after the probe trial, and mice were tested 4 times per day for 3 days for their ability to locate the platform in the opposite quadrant. Trials were recorded with a video camera placed above the center of the pool and the analysis was automated through the ANY-maze™ video tracking system.

#### Radial arm maze (RAM)

The RAM task was carried out as previously described (Manns et al., 2010). Mice were maintained at 85% of their free feeding weights. Each of the 8 radial arms (7 × 38 cm) was baited at its extremity with a food pellet. After placing the mouse in the central area (18.5 cm diameter), the trap doors surrounding the center were opened and the animal was allowed to enter one arm, after which all other doors were closed. When the mouse returned to the center, all doors stayed closed during an inter-trial period of 5 s. A trial is completed once the mouse has collected all food pellets. Re-entries into already visited arms are considered working memory errors. Mice were tested daily until they reach 80% correct choices during three consecutive days.

#### Visual cliff

The test consists of a transparent plastic box placed in a table with the four edges emerging about 20 cm above the top of the table and a rod running along the middle, separating the box into two sections: one has a checker paper placed on the top surface of the box, whereas the other has the same checker paper placed on the bottom surface of the box. The mouse was placed on the rod and should choose within 5 min to walk on the safe side, where the checker paper is on the top surface of the box or the cliff zone (“fake cliff”) where the checker paper is on the bottom surface of the box. Each mouse is tested for 10 trials with the apparatus turned 180° after five trials. Results are expressed in percentages of chosen zones for each mouse.

Statistics were done using SPSS Software, v18.0. One or Two-Way analysis of variance (ANOVA) followed by Bonferroni *post-hoc* comparisons was used to compare the mean differences with multiple factors, i.e., between groups (genotypes), and/or intervals (days), as appropriate. Unpaired Student *t*-test was used when only two groups were compared, unless specified otherwise. Equality of variances was assessed by Levene's Test. Statistical significance was considered only when *p* < 0.05.

### Electrophysiology

After anaesthetization with CO_2_, male mice (3–4 months old) were decapitated and the brains were quickly removed into artificial cerebrospinal fluid (aCSF) containing (in mM): 124 NaCl, 3.7 KCl, 1.2 KH_2_PO_4_, 1 MgSO_4·_7H_2_O, 2.5 CaCl_2·_2H_2_O, 24.6 NaHCO_3_, and 10 D-glucose, maintained at 2–4°C. The pH of solution was between 7.3 and 7.4 when bubbled with 95% O_2_ and 5% CO_2_. 400 μm-thick transverse hippocampal slices were quickly prepared from the right hippocampus using a manual tissue chopper, transferred onto nylon net in an interface chamber (Scientific Systems Design, Canada) and incubated at 32°C at an aCSF flow rate of 1 ml/min and carbogen consumption of 16 l/h. Slices were incubated for at least 3 h in the chamber before starting the experiments. In all experiments, 2 monopolar, lacquer-coated, stainless-steel electrodes (5 MΩ; AM Systems, Carlsborg) were positioned within the stratum radiatum of the CA1 region; one to stimulate the Schaffer collaterals and another to record the fEPSP responses from the apical dendrites. The signals were amplified by a differential amplifier (Model 1700, AM Systems), digitized and monitored online with a custom software. After the incubation period, an input–output curve (afferent stimulation vs. fEPSP slope) was plotted and the test stimulus intensity for each slice was set to elicit a fEPSP slope 40–50% of its maximal response. Test stimulation consisted of four 0.2 Hz biphasic constant-current pulses (0.1 ms/polarity) delivered every 5 min. In all experiments, at least 30 min of stable baseline was recorded before LTP induction. The strong tetanization protocol (STET) consisted of three trains of high frequency stimulation (each train at 100 Hz, 100 biphasic constant-current pulses, single burst, 0.2 ms pulse duration) delivered with an inter-train interval of 10 min (Sajikumar et al., 2005). Theta-burst stimulation (TBS) consisted of 50 bursts (4 stimuli/burst) at an inter-stimulus interval of 10 ms. The 50 bursts were applied over a period of 30 s at 5 Hz (or at an inter-burst interval of 200 ms) (Huang and Kandel, 2005). The initial slopes of fEPSPs were expressed as percentages of baseline averages. The time-matched, normalized data were averaged across replicate experiments and expressed as mean ± SEM. Statistical analyses were performed with GraphPad Prism 6.0.

### Western blotting

Three month old mouse brains were dissected and homogenized using a Glass/Glass dounce homogenizer. The homogenate was spun at 1000 g to remove cell debris and lysed with RIPA buffer [50 mM Tris-HCl pH = 8, 150 mM NaCl, 1 mM EDTA, 1% NP-40, 0.1% SDS, complete protease inhibitors, and phosphatases inhibitors (Roche)]. Lysates were then cleared by centrifugation. For immunoblotting 20–40 μg of total protein was loaded. Fractions were then analyzed by SDS-PAGE and immunoblotted using Abs against STIM1 (ProSci, 4119), STIM2 (Cell Signaling, 4917S), GluA1 (Millipore, MAB2263), pSer845-GluA1 (Millipore, 04-1073), CREB (Cell Signaling, 9197), pSer133-CREB (Cell Signaling, 9191), Cav1.2 (Alomone Labs, ACC-003), pSer1928-Cav1.2 (LifeSpan Bioscience, LS-C145147) and GAPDH (Millipore, MAB374). Immunoblots were developed using horseradish peroxidase (HRP)-conjugated secondary Abs (Jackson Laboratories), followed by detection with enhanced chemiluminescence (ECL, Pierce). Exposure was adjusted to prevent saturation of the ECL signal.

## Results

### Generation of brain-specific *Stim1, Stim2*, and double *Stim1/Stim2* cKO mice

To conditionally inactivate the *Stim* genes in the forebrain, we crossed mice homozygous for *loxP*-flanked (floxed, fl) *Stim1*−, *Stim2*− or *Stim1/Stim2*− alleles (known as *Stim1^fl/fl^*, *Stim2^fl/fl^*, or *Stim1^fl/fl^*/*Stim2^fl/fl^*) (Oh-Hora et al., 2008) with *CaMKIIα-Cre* transgenic mice (Tsien et al., 1996a). Floxed *Stim* males heterozygous for Cre (*Stim^fl/fl^*;*CaMKIIα-Cre^+/−^*) were then mated with *Stim^fl/fl^*;*CaMKIIα-Cre^−/−^* females to produce litters consisting of *Stim^fl/fl^*;Cre^+/−^ (*Stim* cKO) and *Stim^fl/fl^*;Cre^−/−^ (control littermates). Crossing *CaMKIIα-Cre* mice to a conditional tdTomato Cre reporter line Ai9 (Madisen et al., 2010) showed that Cre recombinase is turned on around 1 month after birth and is expressed in virtually all CA1 hippocampal neurons after 90 days (Figure S1A in Supplementary Material).

**Figure 1:**
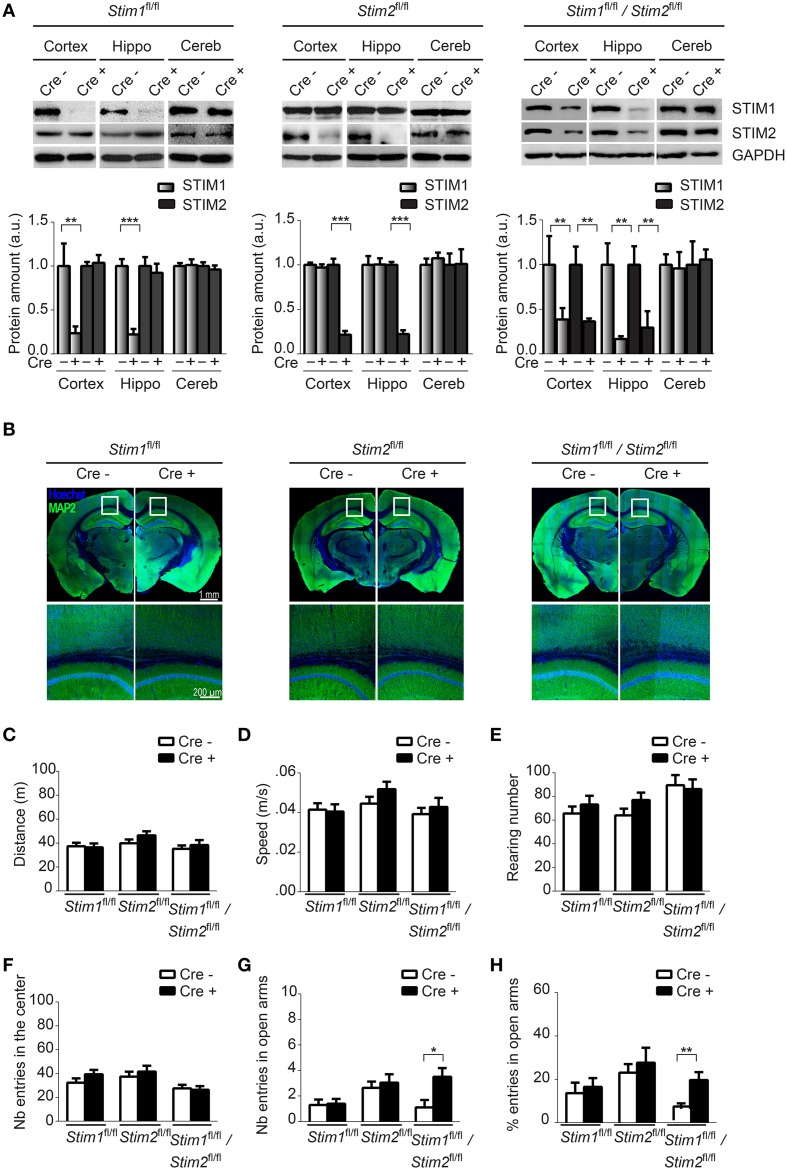
**Characterization of forebrain-specific** ***Stim*** **cKO mouse strains**. **(A)** Upper panels. Immunoblots of STIM1 and STIM2 from cortex, hippocampus (hippo), and cerebellum (cereb) harvested from adult *Stim1^fl^*^/^*^fl^;Cre^+^* (cKO) and *Cre^−^* (control), *Stim2^fl^*^/^*^fl^;Cre^+^* and *Cre^−^*, and double *Stim1^fl/fl^/Stim2^fl^*^/^*^fl^;Cre^+^* and *Cre^−^* mice. Lower panels. Quantification of STIM1 and STIM2 abundance in the *Stim* lines indicated on top. 6 brains were analyzed for each genotype. Data are normalized to *Cre^−^* controls. Blot exposures were adjusted for optimal comparison of Cre^−^ and Cre^+^ groups and thus do not accurately reflect relative abundance of the STIM isoforms in different brain regions. Means ± SD are shown. ^**^p < 0.01 and ^***^p < 0.001, *t*-test. **(B)** Coronal sections of adult mice (4 months old) immunostained for MAP2 (a neuronal marker) and nuclei (Hoechst). Lower panels are a magnification of the area indicated in the upper panels and show the neuropil in part of the cortex and CA1 region of the hippocampus. **(C–F)** Locomotor activity probed by the open field test. **(C)** distance traveled, **(D)** average speed, **(E)** rearing, and **(F)** propensity to visit the center of the field for *Stim1^fl/fl^;Cre^+^* and *Cre^−^*, *Stim2^fl/fl^;Cre^+^* and *Cre^−^* and *Stim1^fl/fl^/Stim2^fl^*^/^*^fl^;Cre^+^* and *Cre^−^* mice. *n* = 10 for each group. Means ± SEM are displayed. There is no significant difference across all genotypes. Number **(G)** and percentage **(H)** of entries in open arm of the elevated plus maze for *Stim1^fl/fl^;Cre^+^* (*n* = 10) and *Cre^−^* (*n* = 10), *Stim2^fl/fl^;Cre^+^* (*n* = 22) and *Cre^−^* (*n* = 22) and *Stim1^fl/fl^/Stim2^fl^*^/^*^fl^;Cre^+^* (*n* = 10) and *Cre^−^* (*n* = 10) mice. Means ± SEM are shown. ^*^p < 0.05, ^**^p < 0.01.

To confirm Cre-mediated excision of the *Stim* genes in these three cKO strains, we analyzed expression of the STIM proteins in different brain regions harvested from adult animals using isoform-specific Abs against STIM1 and STIM2. STIM1 and STIM2 levels were markedly reduced in *Stim1* and *Stim2* cKO mice respectively, in both the hippocampus and cortex, when compared to their control littermates (Figure 1A). As expected, no Cre-mediated excision was observed in the cerebellum. Importantly, ablation of a single *Stim* isoform led to no compensatory increase in the expression of its paralogue. Finally both *Stim* genes were efficiently knocked out in the double *Stim1/Stim2* cKO mouse. Residual expression of the STIM proteins in these cKO lines likely reflects the presence of inhibitory neurons and/or glial cells in the dissected tissues, in which CaMKIIα-Cre is not expressed (Sik et al., 1998). Immunocytochemistry of brain slices revealed no obvious anatomical defects or decrease in neuronal density in *Stim1*, *Stim2* or *Stim1/Stim2* cKO mice up to 4 months of age (Figure 1B, we have not analyzed older animals).

### Ablation of the *Stim* genes does not affect locomotor behavior

To begin to examine the behavior of these *Stim* cKO mice, we subjected them to the open field test (OFT) which assesses general locomotor activity (Stanford, 2007). We observed no significant difference in distance traveled (Figure 1C), average speed (Figure 1D), rearing (vertical activity, Figure 1E), immobility (Figure S1B) and grooming (Figure S1C) between the three *Stim* cKO lines and their control littermates or across genotypes. Neither did we detect any changes in time spent in the center (and periphery, data not shown) of the field (Figure 1F), which has been proposed to reflect exploratory and anxiety-related behaviors (Prut and Belzung, 2003). As another popular model for anxiety-like behavior, we used the elevated plus maze (EPM) which is based on rodents' aversion for open spaces (Carobrez and Bertoglio, 2005). The percentage of entries in the closed and open arms was not significantly different in *Stim1* and *Stim2* cKO mice, relative to their control littermates. The double *Stim1/Stim2* cKO mice, however, spent more time in the open arms than their control littermates, suggesting an increased willingness for exploration and reduced anxiety-like behavior (Figures 1G,H). Together, these results reveal no locomotor defects in these three mutant mice and a higher tendency for exploratory behavior (reduced anxiety) in the double *Stim1/Stim2* cKO mice apparent in the EPM.

### Impaired spatial reference memory in the double *Stim1/Stim2* cKO mice

**Figure 2:**
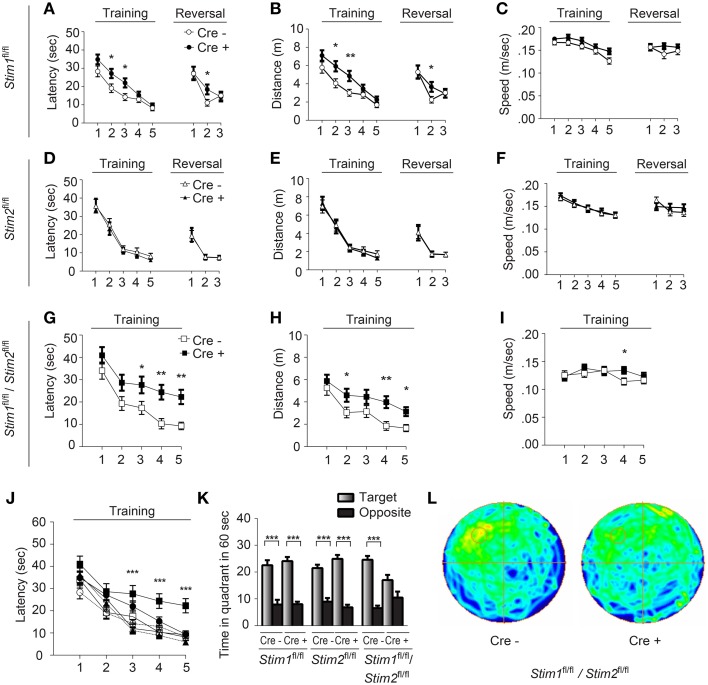
***Stim1/Stim2*** **cKO mice show severe spatial reference memory deficit in the Morris water maze**. Latency to find the escape platform **(A,D,G,J)**, distance **(B,E,H)**, and speed **(C,F,I)** plotted for each training day, or during spatial reversal, for **(A–C)** *Stim1^fl/fl^;Cre^+^*(*n* = 18) and *Cre^−^* (*n* = 14), **(D–F)** *Stim2^fl/fl^;Cre^+^*(*n* = 10) and *Cre^−^* (*n* = 10), and **(G–I)** *Stim1^fl/fl^/Stim2^fl^*^/^*^fl^;Cre^+^* (*n* = 10) and *Cre^−^* (*n* = 10) mice. Means ± SEM are shown. ^*^p < 0.05, ^**^p < 0.01, ANOVA comparison for each day. **(J)** Latency to find platform for each group. Symbols as in **(A,D,G)**. Mean ± SEM are shown. ^***^p < 0.001, ANOVA. **(K,L)** Probe trial. **(K)** Time spent in the target and opposite quadrants. Means ± SEM are shown. ^***^p < 0.001, Two-Way ANOVA. Only target and opposite quadrants are shown. **(L)** Group occupancy plot for *Stim1^fl/fl^/Stim2^fl^*^/^*^fl^;Cre^+^* and *Cre^−^* mice. The platform was located in the upper left quadrant prior to the probe trial.

We next examined spatial learning and memory in these mutant mice in the Morris water maze (MWM). In this spatial reference memory task, the animal is trained to remember the position of a fixed (hidden) escape platform using allocentric spatial cues. Mutant mice and their control littermates received 4 trials a day for 5 consecutive days and performance was assessed by measuring the time (latency) to find the platform. Control mice (across genotypes) performed equally well in this task and reached the platform within 10 sec by the fifth training day (Figures 2A,D,G,J). *Stim1* cKO showed a mild learning defect early on, but performed as well as their control littermates by day 4 (Figures 2A–C). Spatial memory was then analyzed by the probe trial on the 6th day, during which the platform is removed from the pool, and the animal is allowed to swim freely for 60 s. Memory is then inferred based on the time spent in the target quadrant searching for the platform. Both *Stim1* cKO mice and control littermates displayed an equally strong bias toward the target quadrant (Figure 2K), indicating that inactivation of the *Stim1* gene has no effect on the acquisition of spatial memory. *Stim1* cKO mice were also tested for reversal learning, a form of spatial learning which involves extinction of the old platform location and re-learning of its new location. Of interest, *Stim1* cKO mice showed delayed reversal learning compared to their control littermates (Figures 2A,B).

Analysis of the *Stim2* cKO mice in the MWM revealed unaltered performance in spatial learning (Figures 2D–F), probe trials (Figure 2K) and reversal learning (Figures 2D–F). This came as a surprise given the preferential expression of the *Stim2* gene in the hippocampus (Berna-Erro et al., 2009; Skibinska-Kijek et al., 2009; Sun et al., 2014; Garcia-Alvarez et al., 2015) and its recent involvement in excitatory synapse structure and function (Sun et al., 2014; Garcia-Alvarez et al., 2015). In marked contrast, double *Stim1/Stim2* cKO mice exhibited strong deficits in spatial learning and memory. Although learning was not completely abolished in these animals, it was strongly suppressed compared to their control littermates (Figures 2G–I) or all other genotypes tested (Figure 2J). This severe learning impairment persists until the fifth training day and is associated with poor performance on the probe trial (Figures 2K,L). The time the *Stim1/Stim2* cKO mice spent immobile and their average swimming speed during trials were comparable to those of their control littermates (Figure 2I) indicating that this cognitive phenotype is not due to reduced locomotor activity. Likewise, performance in the visual cliff (Figure S2) and behavioral despair tests (i.e., the tail suspension and the forced swim tests, data not shown) argue against a contribution of poor eyesight or lack of motivation to the MWM phenotype. Together these findings show that ablation of both *Stim* genes in the forebrain results in severe deficits in spatial reference memory. The lack of a clear memory phenotype in single *Stim1* or *Stim2* cKO mice suggests redundant or synergistic function of the *Stim* genes in the underlying neural circuits.

### Spatial working memory is not affected in *Stim* cKO mutant mice

**Figure 3:**
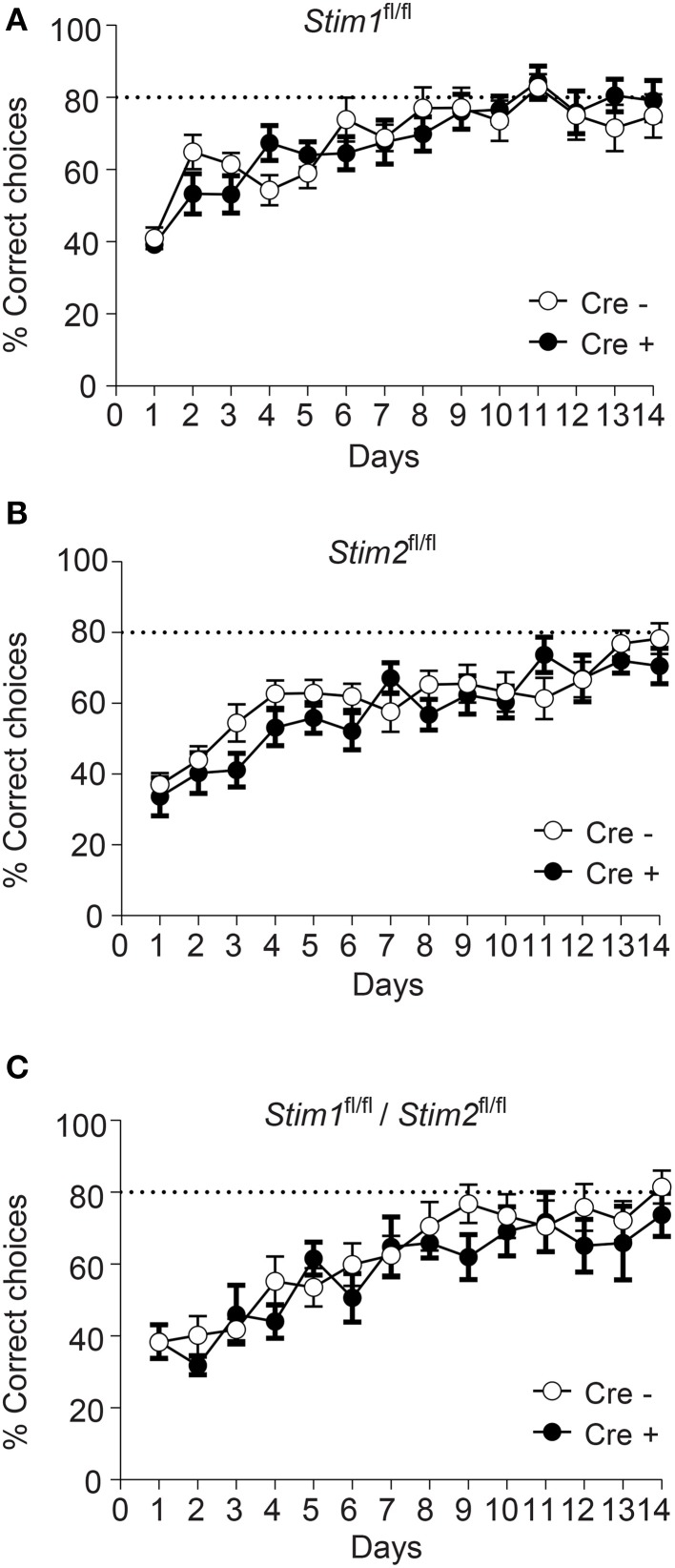
**Unaltered spatial working memory in** ***Stim*** **cKO mutant mice**. **(A–C)** Percentage of correct choices in the radial arm maze plotted for each training day as the mean ± SEM. Performance reaches a plateau at 80% correct choices (dashed line) around the 10th day of training. **(A)** *Stim1^fl/fl^;*Cre*^+^* (*n* = 12) and *Cre^−^* (*n* = 12) mice. **(B)** *Stim2^fl/fl^;Cre^+^*(*n* = 12) and *Cre^−^* (*n* = 12) mice. **(C)** *Stim1^fl/fl^/Stim2^fl^*^/^*^fl^;Cre^+^* (*n* = 7) and *Cre^−^* (*n* = 10) mice. Two Way ANOVA showed a learning effect (*p* < 0.001 with “days” as within factor) but no genotype effect (*p* > 0.05, “genotype” as in between factor).

We next evaluated the performance of *Stim* cKO mutant mice in the radial arm maze (RAM), which assesses a form of short-term, hippocampus-dependent, spatial working memory (Olton, 1979). In contrast to the MWM where the target (hidden platform) is fixed throughout the trials, the correct spatial response in the RAM varies within trials and the animal must use newly-acquired spatial information for an optimal reward strategy. Our RAM set-up consists of eight arms radiating from a central platform. Each arm is baited with a food pellet and food rewards are not replaced within a trial. The animals must therefore keep track of each arm they previously visited for efficient performance. Re-entry into a previously baited arm is defined as a working memory error. Performance of *Stim1*, *Stim2*, *Stim1/Stim2* cKO mice and their control littermates steadily increases to reach a plateau around the 10th trial, at approximately 80% correct choices (about 1.1 error per trial on average). There was no significant difference between *Stim* cKO mice and their control littermates or across genotypes (Figures 3A–C). Collectively, these findings support the existence of distinct forms of spatial memory (e.g., short-term versus long-term) governed by different neural algorithms (Reisel et al., 2002; Schmitt et al., 2003; Bannerman et al., 2012) and indicate that the *Stim* genes specifically regulate spatial reference memory.

### Inactivation of both *Stim* genes results in enhanced L-LTP and PKA signaling

**Figure 4:**
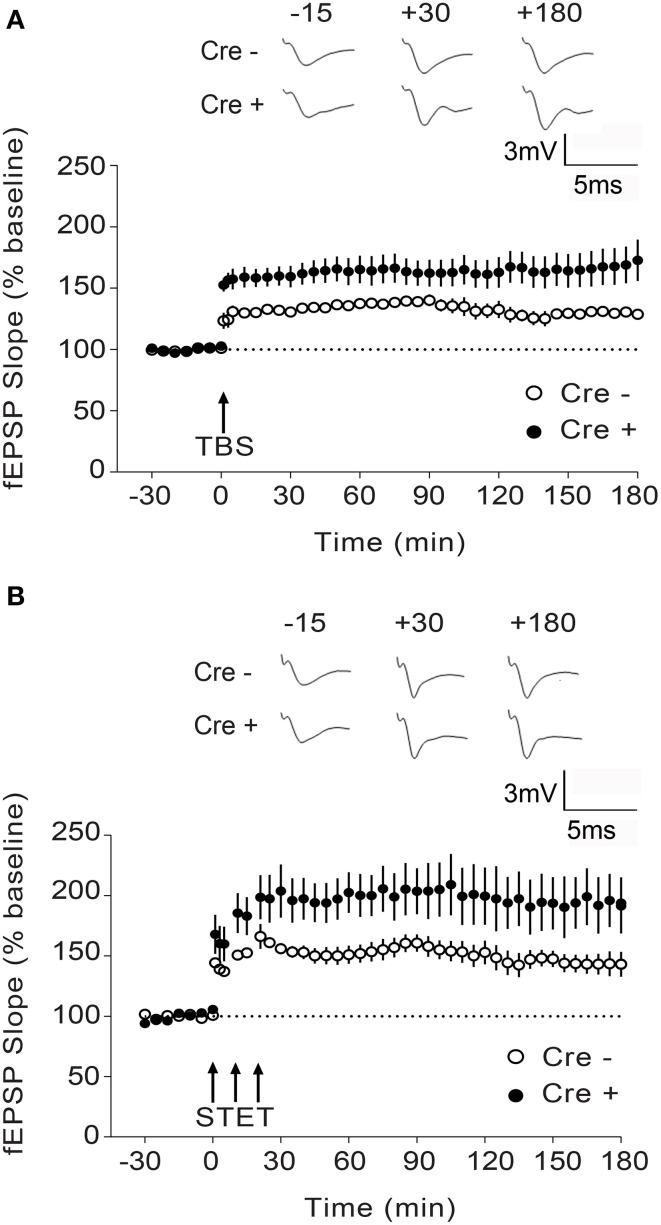
**Enhanced L-LTP in** ***Stim1/Stim2*** **cKO mice**. **(A)** Theta burst (TBS)-induced LTP recorded in CA1 from *Stim1/Stim2; Cre^+^* (*n* = 8 slices from 5 mice) mice and their control (*Cre^−^*) littermates (*n* = 6 slices from 4 mice). Potentiation in both cases is significantly different from the pre-induction values (+180 min, *p* < 0.01; paired *t*-test). By the end of 3 h, mean potentiation of *Cre^+^* group (172.71 ± 16.85%) is significantly different from *Cre^−^* group (128.72 ± 3.65%); Two-sample *t*-test, *p* < 0.05. **(B)** Strong tetanization (STET)-induced LTP recorded in CA1 from *Stim1/Stim2;Cre^+^* (*n* = 6 slices from 4 mice) mice and their control (*Cre^−^*) littermates (*n* = 7 slices from 3 mice). Potentiation in both the groups is significantly different from the pre-induction values (+180 min, *p* < 0.01; paired *t*-test). By the end of 3 h, mean potentiation of *Cre^+^* group (194.75 ± 21.13%) is significantly different from *Cre^−^* group (143.12 ± 9.16%); Two-sample *t*-test, *p* < 0.05. The insets show representative fEPSP traces at −15, +30, and +180 min before and after stimulation. Data are plotted as mean ± SEM.

To determine whether the *Stim* genes regulate synaptic plasticity in hippocampal circuits, we measured long-term potentiation (LTP) at CA3-CA1 hippocampal synapses. LTP was elicited by high-frequency stimulation of the Schaffer collaterals and recorded by measuring field EPSPs (excitatory postsynaptic potentials) in the CA1 region. We chose to elicit LTP using two stimulation paradigms, TBS (theta burst stimulation) and STET (strong tetanus), both of which induce a long-lasting, protein synthesis-dependent form of LTP (late-phase LTP or L-LTP) (Huang and Kandel, 2005; Sajikumar et al., 2005), and we restricted our analysis to the double *Stim1/Stim2* cKO mice, since they display spatial memory deficits. To our surprise, both TBS- and STET-induced L-LTP were markedly enhanced in the double *Stim1/Stim2* cKO mice compared with their control littermates (Figures 4A,B). Thus, L-LTP and spatial reference memory are inversely correlated in *Stim1/Stim2* cKO mice.

While most studies report a positive correlation between LTP and spatial memory (reviewed in Silva, 2003), dissociation between LTP and distinct forms of spatial memory has been observed in several different mouse strains (Zamanillo et al., 1999; Kaksonen et al., 2002; Pineda et al., 2004; Rutten et al., 2008). Notably, enhanced LTP and impaired spatial memory has been reported in mice with targeted deletion of genes downregulating adenylate cyclase activity or cAMP levels, resulting in an overall increase in cAMP/PKA signaling (Pineda et al., 2004; Rutten et al., 2008). This, together with data from our own laboratory identifying a central role of STIM2 in cAMP/PKA-dependent phosphorylation of AMPA receptors (Garcia-Alvarez et al., 2015), prompted us to examine PKA signaling in *Stim* cKO mice. We quantified phosphorylation of three central regulators of synaptic plasticity and memory, namely the AMPAR subunit GluA1, CREB (cAMP response element-binding protein) and the L-type voltage-dependent Ca^2+^ channel Cav1.2, on serine residues that are known PKA targets (De Jongh et al., 1996; Silva et al., 1998; Esteban et al., 2003).

**Figure 5:**
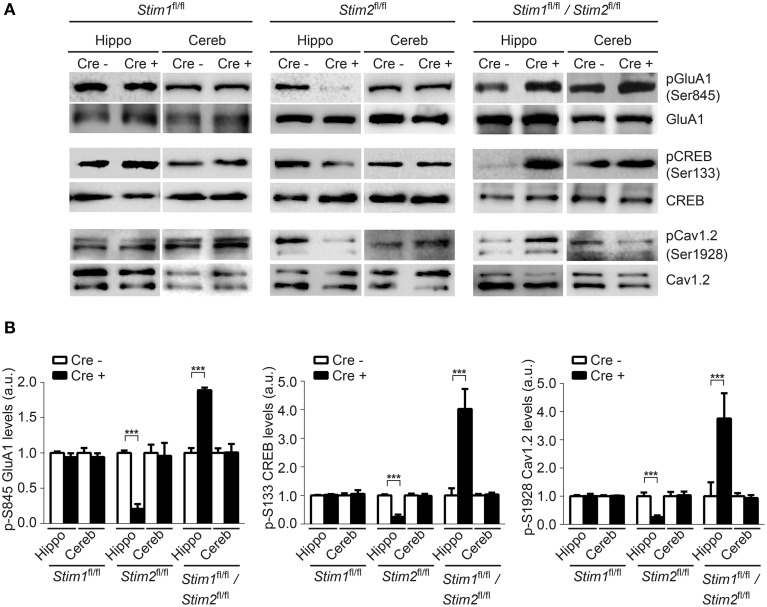
**Effect of the** ***Stim*** **genes on PKA signaling**. **(A)** Phospho-immunoblots of pSer845-GluA1, pSer133-CREB, and pSer1928-Cav1.2 in hippocampus (hippo) and cerebellum (cereb) harvested from the three *Stim* cKO (Cre^+^) lines and their control (*Cre^−^)* littermates. **(B)** Quantification of pSer845-GluA1, pSer133-CREB, and pSer1928-Cav1.2 levels (*n* = 3 for each group). Phospho-protein signals are normalized within each *Stim* genotype to the *Cre*^−^ group. Blot exposures were adjusted for optimal comparison of phospho-signals in Cre^−^ and Cre^+^ groups and thus do not accurately reflect relative levels of phosphoproteins in the different *Stim* lines. Means ± SD are shown. ^***^p < 0.001, *t*-test.

Phosphorylation of GluA1 (pSer-845), CREB (pSer-133) and Cav1.2 (pSer1928) in the hippocampus was unchanged in *Stim1* cKO mice, but was markedly reduced in *Stim2* cKO mice (Figures 5A,B), consistent with our previous findings in primary neuron cultures (Garcia-Alvarez et al., 2015). By contrast, phosphorylation of these three proteins was strongly up-regulated in the hippocampus of double *Stim1/Stim2* cKO mice. Although, we cannot exclude the possible involvement of other kinases in STIM-dependent regulation of CREB and Cav1.2 phosphorylation (Silva et al., 1998; Yang et al., 2005), reciprocal regulation of GluA1 Ser-845 phosphorylation in *Stim2* and *Stim1/Stim2* cKO mice is indicative of a complex, synergistic interaction of the STIM isoforms in regulating PKA signaling (see Section Discussion). Increased phosphorylation of GluA1, CREB, and Cav1.2 is associated with elevated activity of these proteins (De Jongh et al., 1996; Silva et al., 1998; Esteban et al., 2003) and is likely to contribute to the enhanced LTP observed in the double *Stim1/Stim2* cKO mice. Together, these findings show that the *Stim* genes are key regulators of phosphorylation and synaptic plasticity in neural circuits underlying spatial memory.

## Discussion

### The *Stim* genes are required for a specific form of long-term spatial memory

We report here the first behavioral analysis of mutant mice with conditional deletion of the *Stim* genes in the forebrain. *Stim2* cKO mice show no apparent defect in spatial reference memory (MWM), spatial working memory (RAM) or in any of the other behavioral tests we conducted. These results are in apparent contradiction with an earlier study reporting impaired performance of *Stim2^−/−^* null mice in the MWM (Berna-Erro et al., 2009). *Stim2^−/−^* null mice, however, die after 8 weeks (Berna-Erro et al., 2009) or even earlier (4–5 weeks, Oh-Hora et al., 2008), which complicates the interpretation of learning/memory tasks, as poor performance in these tasks could result from pleiotropic effects on development/health, rather than a specific function of the *Stim2* gene in circuits encoding memory.

Forebrain-specific inactivation of *Stim1* has a subtle impact on spatial learning and memory. Spatial learning is delayed in the *Stim1* cKO mice in early trials of the MWM, although by the fifth trial, performance is similar to their control littermates. In addition, spatial reversal in the MWM is impaired in these mutant mice, a phenotype recently described in a mouse strain with conditional ablation of the NMDA receptor subunit *Grin1* (GluN1) in the dentate gyrus and CA1 regions of the hippocampus (Bannerman et al., 2012). Decreased performance in spatial reversal reflects an inability to learn a new location in a familiar environment and distinguish between two competing or overlapping memories, a phenomenon akin to pattern separation (Bannerman et al., 2014).

Inactivation of both *Stim* genes in the forebrain resulted in a severe deficit in spatial reference memory in the MWM. Memory impairment in the double *Stim1/Stim2* cKO mice is comparable in extent to that seen in floxed GluN1 mice crossed to the same Cre driver line (Tsien et al., 1996b). This, together with the absence of any detectable anatomical defects in *Stim1/Stim2* cKO mice suggests that the STIM proteins regulate key aspects of synaptic function/plasticity in neural circuits encoding spatial memory.

Of interest, ablation of both *Stim* genes has no effect on spatial working memory (RAM) suggesting a specific impact of the *Stim* genes on neural circuits encoding spatial reference memory. Dissociation between spatial reference memory and spatial working memory has been observed before, in mouse strains with genetic ablation of glutamate receptors. GluA1 null mice show impaired spatial working memory in the RAM (Reisel et al., 2002) but exhibit intact spatial reference memory in the MWM. Similarly, mice with targeted deletion of GluN1 in dentate gyrus/CA1 show selective impairment of spatial working memory (Bannerman et al., 2012). Our findings therefore support the idea that spatial memory is not a single process, but instead, takes on distinct forms that can be genetically disentangled.

### Dual role of the *Stim* genes in cognition and emotion

Impaired performance of *Stim1/Stim2* cKO mice in the MWM and their reduced anxiety-like behavior in the EPM suggest that the *Stim* genes impact circuits regulating both cognition and emotion. Lesion studies, gene expression analyses and anatomical connectivity of the hippocampus have led to a model describing its functional parcellation into at least two sub-regions (Fanselow and Dong, 2010). The dorsal hippocampus (DH) appears to be specifically involved in spatial and declarative memories (Moser et al., 1995), while the ventral hippocampus (VH) seems to preferentially control emotional behaviors, in particular those associated with negative affect, like anxiety or fear (Kjelstrup et al., 2002; Maren and Holt, 2004). Of interest, lesion of the VH (Mchugh et al., 2004), or inactivation of the NMDA receptor subunit GluN1 in the dentate gyrus (Niewoehner et al., 2007) both result in increased time spent in the open (anxiogenic) arms of the EPM, a phenotype that we also observed in the *Stim1/Stim2* cKO mice. Based on these findings and the expression of *Stim1* and *Stim2* throughout the dorso-lateral axis of the hippocampus (The Allen mouse brain Atlas, Lein et al., 2007), we speculate that the *Stim* genes regulate both dorsal and ventral functions of the hippocampus.

### LTP and spatial memory

LTP (or an LTP-like process) is widely considered as the dominant cellular mechanism underlying experience-dependent changes in synaptic connections and information storage. Many of its characteristics (associativity, cooperativity, and temporal resilience) are consistent with a role in memory formation and maintenance. In addition, a massive body of literature (reviewed in Silva, 2003) suggests that LTP and hippocampus-dependent forms of memory rely on the same cellular pathways and correlate with one another. For example, ablation of the GluN1 subunit of the NMDAR in the forebrain obliterates both LTP and spatial memory (Tsien et al., 1996b), while overexpression of GluN2B has the opposite effect (Tang et al., 1999). This has led to the idea that manipulations boosting LTP may have therapeutic potential for cognitive enhancement. However, an increasing number of studies, relying on different genetically-modified mouse strains, also report clear dissociation of LTP and memory, thereby challenging the causal relationship between these two processes (Meiri et al., 1998; Migaud et al., 1998; Zamanillo et al., 1999; Pineda et al., 2004; Rutten et al., 2008; Kim et al., 2009; Dilekoz et al., 2015). Relevant to our work, genetic manipulations of key plasticity-regulating genes, such as PSD-95 (Migaud et al., 1998), IRSp53 (Kim et al., 2009), PTPδ (Uetani et al., 2000), and components of the cAMP signaling pathway (Pineda et al., 2004; Bourtchouladze et al., 2006; Rutten et al., 2008; Viosca et al., 2009) have been shown to impair spatial memory while, at the same time, enhance LTP. Our findings therefore further support the view that enhancement of LTP in the hippocampus does not necessarily translate into superior learning and memory functions. One possible interpretation of these data is that abnormally high synaptic potentiation alters synaptic plasticity and disrupts input-specific learning rules in the hippocampus (see Pineda et al., 2004). In support of this model, enhanced LTP results in abnormal place cell activity (Taverna et al., 2005) and saturation of LTP impairs spatial learning (Moser et al., 1998). Additionally, mathematical simulations of neural networks indicate that synaptic potentiation must be proportionally constrained by synaptic depression for optimal information storage (Sejnowski, 1977; Dayan and Willshaw, 1991; Migaud et al., 1998), suggesting that uncontrolled LTP may negatively impact network performance.

### Synergistic regulation of PKA signaling by the *Stim* genes

cAMP/PKA signaling regulates multiple aspects of neuronal and synaptic plasticity and is crucial for learning and memory in both invertebrates and vertebrates (reviewed in Silva et al., 1998; Kandel, 2001). Proper dosage of cAMP signaling appears to be central for information storage, as genetic mutations with both negative and positive effects on this signal transduction pathway can be detrimental to memory formation (Livingstone et al., 1984; Skoulakis et al., 1993; Pineda et al., 2004).

We have previously demonstrated an essential role of STIM2 in mediating PKA phosphorylation of GluA1 (Garcia-Alvarez et al., 2015). Results described in the present study confirm involvement of the *Stim* genes in regulating phosphorylation of PKA targets *in vivo*, but also reveal a complex interaction of these genes in controlling PKA signaling in the hippocampus. Indeed, PKA phosphorylation of GluA1 on Ser-845 is markedly upregulated in *Stim1/Stim2* cKO mice, but is instead repressed or unaffected in *Stim2* and *Stim1* cKO mice respectively. The same reciprocal pattern is observed for pSer1928-Cav1.2 and pSer133-CREB, although it is possible that STIMs regulate phosphorylation of these sites through kinases other than PKA (Silva et al., 1998; Yang et al., 2005).

The impact of single and double gene ablation on PKA signaling implies the presence of synergy between the *Stim* genes (i.e., the contribution of both gene deletions to the PKA phenotype far exceeds the expected additive effects of individual gene deletions). A recent survey suggests that synergy often arises from functionally redundant gene pairs, particularly among signaling genes, providing increased adaptability, robustness and evolvability (Kafri et al., 2009). Such a synergistic interaction between the *Stim* genes could be important for fine-tuning PKA activity and synaptic plasticity.

The effect of the *Stim* genes on phosphorylation of GluA1, CREB and Cav1.2 is likely to influence synaptic plasticity and behavior in multiple ways. PKA phosphorylation of GluA1 on Ser-845 promotes surface delivery and synaptic recruitment of the AMPAR during LTP (Esteban et al., 2003; Oh et al., 2006). Increased pSer-845 of GluA1 in *Stim1/Stim2* cKO mice is thus likely to contribute to the observed enhancement in the induction of LTP. Similarly, elevated levels of pSer1928-Cav1.2 and pSer133-CREB could promote L-LTP in the *Stim1/Stim2* cKO mice, as both Cav1.2 and CREB are necessary for protein synthesis-dependent LTP in the hippocampus and long-term spatial memory (Bourtchuladze et al., 1994; Moosmang et al., 2005).

In addition to the proposed role of the STIM proteins in regulating PKA signaling outputs, STIMs are also critical regulators of Ca^2+^ homeostasis. The aberrant synaptic potentiation and behavior observed in the double *Stim1/Stim2* cKO mice could therefore also be linked to altered ER Ca^2+^ release (Hartmann et al., 2014) or Ca^2+^ influx (Sun et al., 2014) at the synapse.

In conclusion, we identified novel functions of the *Stim* genes in shaping excitatory circuits underlying spatial memory in the mouse brain. These findings, together with a handful of recent papers (Hartmann et al., 2014; Sun et al., 2014; Garcia-Alvarez et al., 2015) point to an emerging role of ER-to-synapse signaling in the mammalian nervous system, and are of immediate relevance to synaptic disorders associated with ER dysfunction.

## Author contributions

ZB and GG performed the behavioral analyses. MS performed the electrophysiology experiments. GG, BL, and KY did the biochemistry and immunohistochemistry experiments. MO generated the *Stim* cKO mice. ZB, SS, and MF designed and supervised the project. MF wrote the manuscript.

### Conflict of interest statement

This work is partly funded by a joint grant from the Agency of Science Technology and Research (Astar, Singapore) and Janssen Pharmaceuticals, Inc. Janssen Pharmaceuticals had no involvement in any aspect of study design, data collection, analysis and interpretation, or decision to submit this manuscript for publication. The authors declare that the research was conducted in the absence of any commercial or financial relationships that could be construed as a potential conflict of interest.

## Supplementary material

Figure S1 **(A)** tdTomato expression in CA1 pyramidal neurons in the tdTomato Cre reporter line (Ai9) crossed to *CaMKIIa-Cre^+^* mice, atP30 and P90. Nuclei were stained with Hoechst. **(B)** Immobility and **(C)** grooming data derived from the open field test for each of the *Stim* cKO lines and control littermates.

Figure S2 **Visual cliff**. Percentage of safe actions (correct responses) in the cliff test after 10 trials (mean ± SEM) in *Stim1/Stim2;Cre^+^* (cKO) mice (*n* = 7) and control (Cre^−^) littermates (*n* = 10). There was no significant difference between the two groups.

## Abbreviations

STIM: Stromal Interaction Molecule
LTP: long-term potentiation
CREB: cAMP response element-binding protein
PKA: protein kinase A
AMPA: α-Amino-3-hydroxy-5-methyl-4-isoxazolepropionic acid
ER: endoplasmic reticulum
SOCE: store-operated Ca^2+^ entry
OFT: Open Field Test
EPM: Elevated Plus Maze
MWM: Morris Water Maze
RAM: Radial Arm Maze
GluA1: AMPA-type glutamate receptor subunit 1
GluN1: NMDA-type glutamate receptor subunit 1.
