# Supplementary figures and images for: Impaired spatial memory and enhanced long-term potentiation in mice with forebrain-specific ablation of the Stim genes
Source: Front Behav Neurosci. 2015 Jul 14;9:180. doi: 10.3389/fnbeh.2015.00180 (PMC4500926; doi:10.3389/fnbeh.2015.00180)

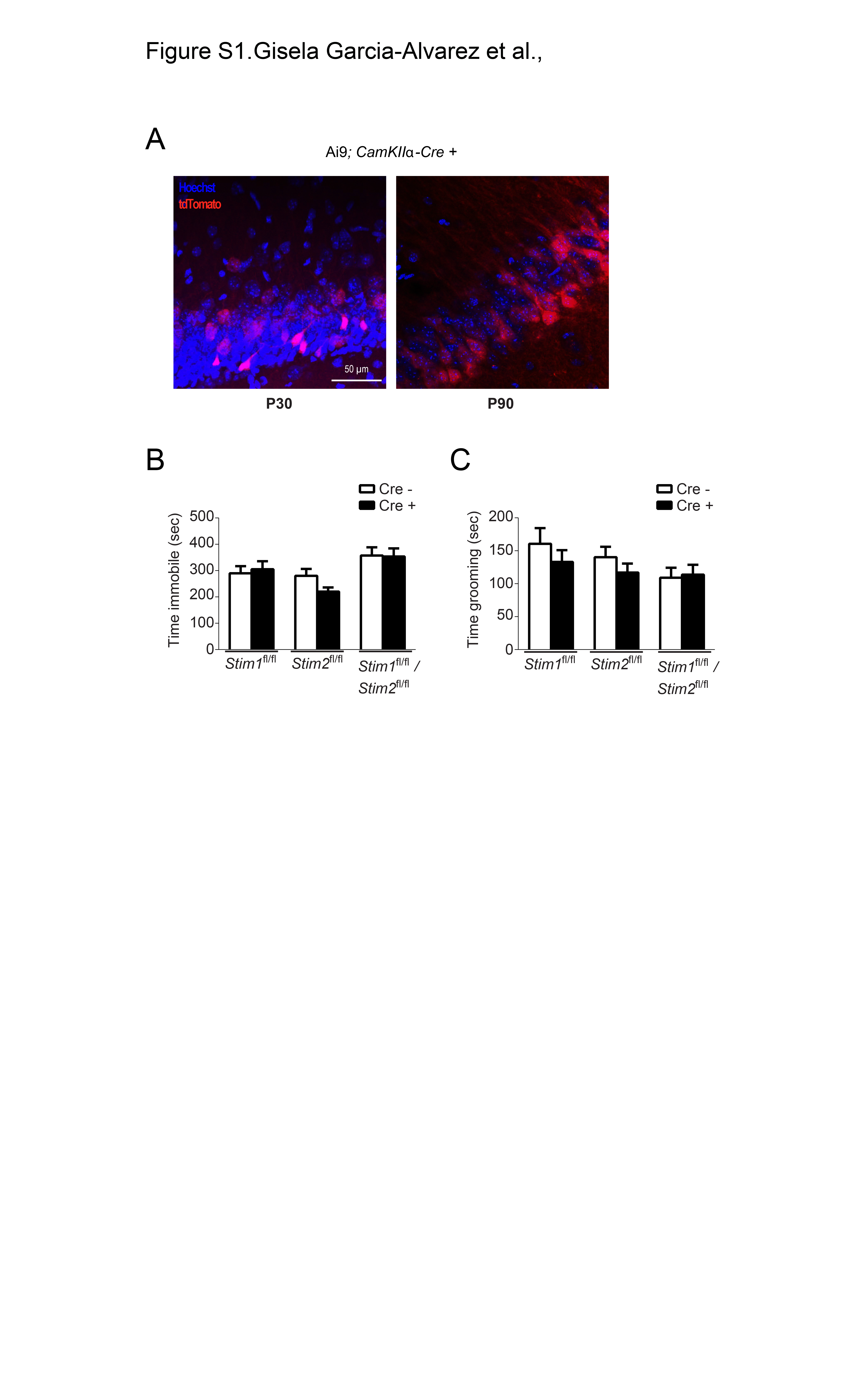

Supplement: Figure S1 [file Image1.TIF]

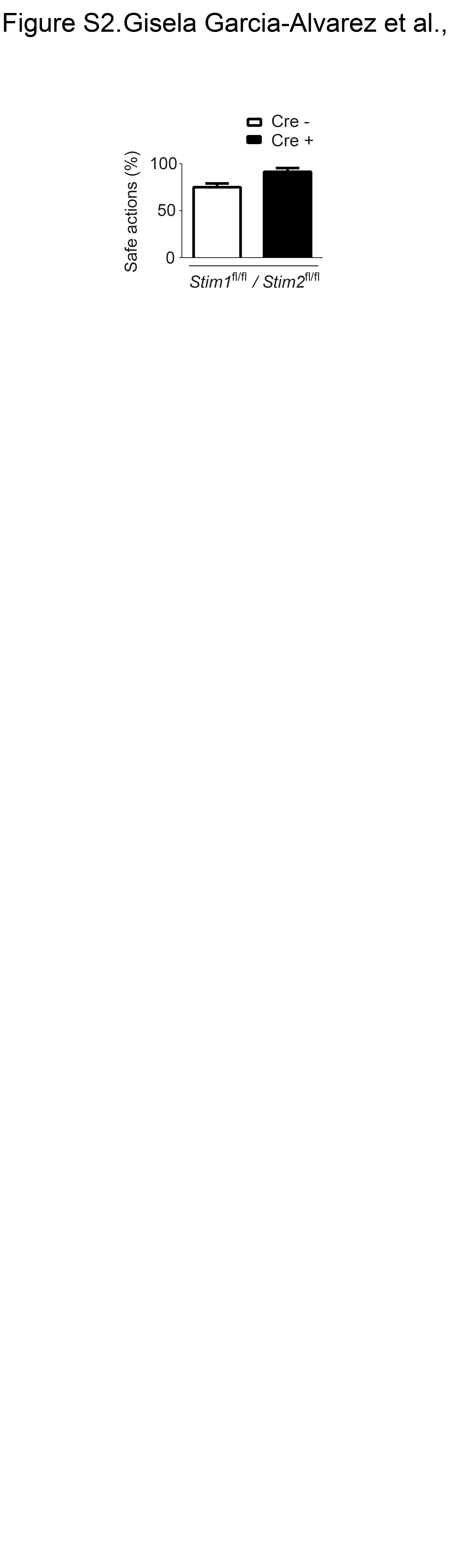

Supplement: Figure S2 [file Image2.TIF]
